# Social preference is maintained in mice with impaired startle reflex and glutamate/D-serine imbalance induced by chronic cerebral toxoplasmosis

**DOI:** 10.1038/s41598-021-93504-1

**Published:** 2021-07-07

**Authors:** Mariana Acquarone, A. Poleto, A. F. Perozzo, P. F. R. Gonçalves, R. Panizzutti, J. R. L. Menezes, G. A. Neves, Helene Santos Barbosa

**Affiliations:** 1 Instituto de Ciências Biomédicas, Universidade Federal do Rio de Janeiro, Rio de Janeiro, Brazil; 2 Laboratório de Biologia Estrutural, Instituto Oswaldo Cruz, Fundação Instituto Oswaldo Cruz, Rio de Janeiro, Brazil

**Keywords:** Cognitive neuroscience, Infection, Neuroscience, Pathogenesis, Diseases, Psychiatric disorders

## Abstract

*Toxoplasma gondii* is an opportunistic protozoan pathogen with a wide geographic distribution. The chronic phase of toxoplasmosis is often asymptomatic in humans and is characterized by tissue cysts throughout the central nervous system and muscle cells. *T. gondii* and other pathogens with tropism for the central nervous system are considered risk factors in the etiology of several neuropsychiatric disorders, such as schizophrenia and bipolar disorder, besides neurological diseases. Currently, it is known that cerebral toxoplasmosis increases dopamine levels in the brain and it is related to behavioral changes in animals and humans. Here we evaluate whether chronic *T. gondii* infection, using the cystogenic ME-49 strain, could induce behavioral alterations associated with neuropsychiatric disorders and glutamatergic neurotransmission dysfunction. We observed that the startle amplitude is reduced in the infected animals as well as glutamate and D-serine levels in prefrontal cortical and hippocampal tissue homogenates. Moreover, we did not detect alterations in social preference and spontaneous alternation despite severe motor impairment. Thus, we conclude that behavioral and cognitive aspects are maintained even though severe neural damage is observed by chronic infection of C57Bl/6 mice with the ME-49 strain.

## Introduction

Currently, it is known that neuropsychiatric disorders are triggered as a consequence of interactions between neurobiological and environmental factors. However, recent studies failed to demonstrate such consistency for a causal relationship^1,2^. In order to highlight this relationship, large-scale studies focused in investigating the impact of infectious agents as environmental components^3^. For instance, it was found that pathogens with tropism for the central nervous system, such as cytomegalovirus^4^, herpes virus^5^, or *Toxoplasma gondii*^4^ are considered important risk factors. The relationship between toxoplasmosis and schizophrenia has been one of the most studied by now, demonstrating high levels of seroprevalence among patients^6–8^. Besides schizophrenia, other neuropsychiatric disorders and neurological diseases have been correlated to toxoplasmosis, such as bipolar disorder^9^, obsessive–compulsive disorder^10,11^, aggressive and suicidal behavior^12,13^, but also Parkinson’s^14^ and Alzheimer’s disease^15^.

*T. gondii* is an opportunistic protozoan pathogen of wide geographic distribution^16^. The acute phase of the disease is symptomatic. In contrast, the chronic phase, characterized by cyst’s presence throughout the central nervous system and muscle cells of the intermediate host, is considered the latent and asymptomatic phase. Once in the brain, T. gondii increases dopamine levels via two encoding tyrosine hydroxylase genes^17^, which presents high similarity with the dopamine-synthesizing enzyme presented in mammals. Such dopaminergic alterations may be related to behavioral changes observed in animals and humans with chronic toxoplasmosis, and have been reported in schizophrenia and bipolar disorder. In fact, dopaminergic hyperfunction is considered partially responsible for psychotic symptoms development and underlies the use of antipsychotic drugs to treat such disorders^18–20^. 

Besides dopaminergic alterations, excitatory glutamatergic neurotransmission is also affected in neuropsychiatric disorders. Animal models for schizophrenia have abnormally increased levels of glutamate, an agonist of the N-methyl-D-aspartate receptor (NMDAR)^21,22^. In addition, mutant animals to serine racemase, a D-serine (NMDAR co-agonist) synthesizing enzyme, have also been considered as working models for the study of schizophrenia^23^. In patients, cerebrospinal fluid and serum analyses confirmed increased glutamate and decreased D-serine levels^24–27^. Such evidence is part of the NMDAR hypofunction hypothesis, according to which lower levels of NMDAR activation might relate to schizophrenia symptoms, especially to cognitive dysfunctions and impaired sensory processing^28^. Interestingly, *T. gondii* infection alters synaptic protein’s composition and it is accompanied by downregulation of glutamatergic receptors^29,30^. These alterations have been related to cognitive deficits and behavioral abnormalities^12,31,32^. However, there is currently no evidence for alterations of NMDAR agonist’s levels within brain areas involved in cognitive functions such as the prefrontal cortex and hippocampus.

Due to the epidemiological relationship between toxoplasmosis and neurological or neuropsychiatric changes, we evaluated whether chronic *T. gondii* infection (using the ME-49 strain) changes critical aspects of behavior associated with neuropsychiatric disorders as social preference, working memory, and sensorimotor gating. We also investigated a possible hypofunction in glutamatergic neurotransmission in the prefrontal cortex and hippocampus by measuring glutamine, glutamate, L-serine and D-serine levels.

## Results

### Chronic *T. gondii* infection impairs body weight gain and induces motor deficits

**Figure 1:**
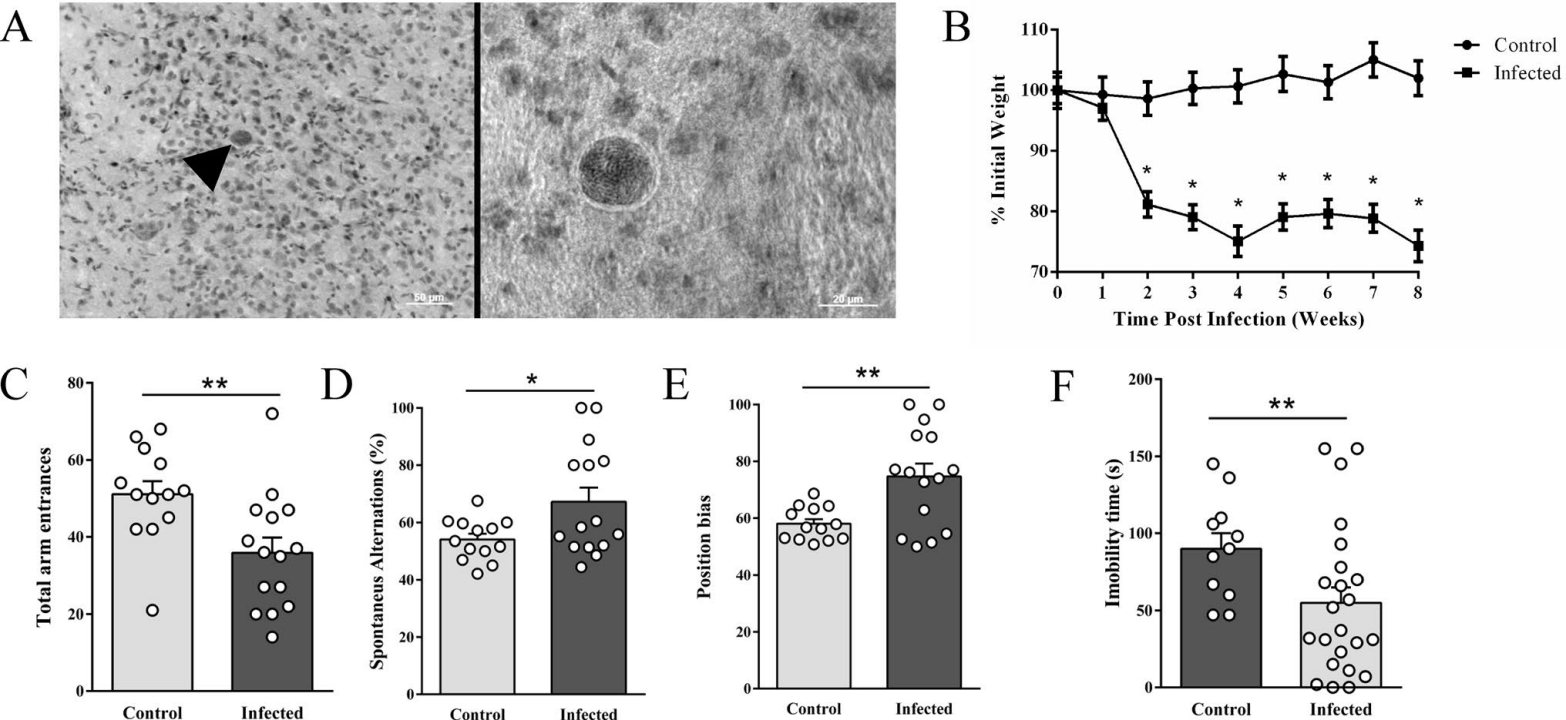
Histophatology, weight loss and motor impairments in chronic *T. gondii* infection. *T. gondii* infected mouse brain section stained with hematoxylin and eosin showing a single large protozoal cyst (arrowhead) with no apparent inflammatory reaction (**A**). Percentage change in body weight of control and infected mice showed reduction in total body weight by *T. gondii* from week post-infection 2 to 8 (**B**). Infection decreased immobility time in the tail suspension test (**C**). In the spontaneous alternations task (Y-maze), a reduction in total arm entrances (**D**), an increase of spontaneous alternations percentage (**E**) as well as the position bias in the infected group (**F**) were indicated. Asterisks indicate statistically significant differences. *p < 0.05 and **p < 0.01. Symbols correspond to individual subjects. Weight percentage comparison: control (n = 13), infected (n = 23). Tail suspension test: control (n = 11), infected (n = 20). Spontaneous alternations task: control (n = 13), infected (n = 15). Scale bar: 50 µm (left); 20 µm (right).

Chronic infection was confirmed by the presence of multifocal *T. gondii* tissue cysts throughout several brain sections (Fig. 1A) and by body weight loss (Fig. 1B). Mice lost around 20% of their total body mass during the acute phase of infection (7 to 14 days post-infection (dpi)), followed by a typical weight loss stabilization by 30 dpi, characterizing the chronic phase. Eight weeks after infection, the animals' behavior was evaluated. They exhibited stereotyped behaviors, including retropulsion, tail dorsiflexion (Straub tail), and circling. At the tail suspension test (Fig. 1C), mice chronically infected presented a significant decrease in the immobility time (55 ± 10 s) when compared to the control group (90 ± 10 s, *p* = 0.038). A qualitative analysis of mice movements in this test showed a high incidence of tail suspension circling (an indicator of neurological damage)^33^ and the presence of hindlimb clasping (an indicator of motor weakness)^34^ in the infected group. Thus, it is likely that the changes in the immobility time presented by infected mice are related to motor or neurological damage and not to a depression-like phenotype.

The motor impairment of chronically infected mice was also evident in the spontaneous alternations (Y maze) task since this group presented a reduction in the total number of arms entries (Fig. 1D, 36 ± 4 entrances) when compared to the control group (51 ± 3 entrances, *p* = 0.008). Infected mice also showed a significant increase in the percentage of spontaneous alternations (Fig. 1E, 54 ± 2% control, 67 ± 5% infected, *p* = 0.028). However, this parameter was influenced by infected mice’s preference to turn to the same side (right or left) when exploring the maze. This preference was confirmed by the position bias index calculation where a statistically significant difference between groups was detected (Fig. 1F, 58 ± 2% control, 75 ± 4% infected, *p* = 0.003).

### Chronic toxoplasmosis significantly decreased startle reflex and PPI in mice

**Figure 2:**
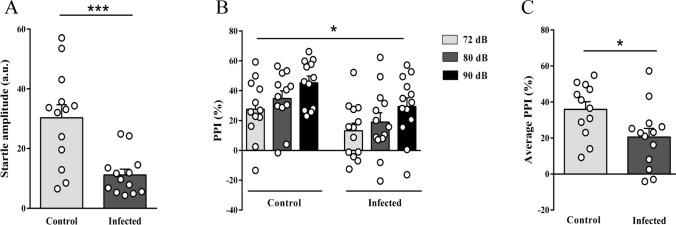
Deficit of startle reflex and prepulse inhibition in chronic *T. gondii* infection. Infected mice showed a significant decrease in startle amplitude (**A**) when 120 dB pulse was utilized without a prepulse. A reduction in %PPI (**B**) and %PPI average (**C**) were observed. Asterisks indicate statistically significant differences. *p < 0.05 and ***p < 0.001. Symbols correspond to individual subjects. dB = decibel. Control (n = 12), infected (n = 13).

In PPI test, the selected pulse intensity (120 dB) induced a startle response in both control and infected mice. However, infected mice showed a significant decrease in startle amplitude (Fig. 2A, 30.3 ± 4.4 a.u. control, 11.2 ± 1.8 a.u. infected, *p* < 0.001). In the PPI evaluation, a significant effect of prepulse intensity was detected (Fig. 2B, *p* = 0.010), where a gradual increase in % PPI as the prepulse intensity increases was observed in both experimental groups (statistical significance achieved for 90 dB *vs*. 72 dB, *p* = 0.007). To this analysis, a significant group effect was also detected (*p* = 0.048), showing that mice with chronic toxoplasmosis presented a significant PPI impairment. This impairment was also detected when the mean % of PPI was analyzed (Fig. 2C, 35.8 ± 4.3% control and 20.5 ± 4.8% infected, *p* = 0.028).

### Chronic *T. gondii* infection did not impair social preference in mice

**Figure 3:**
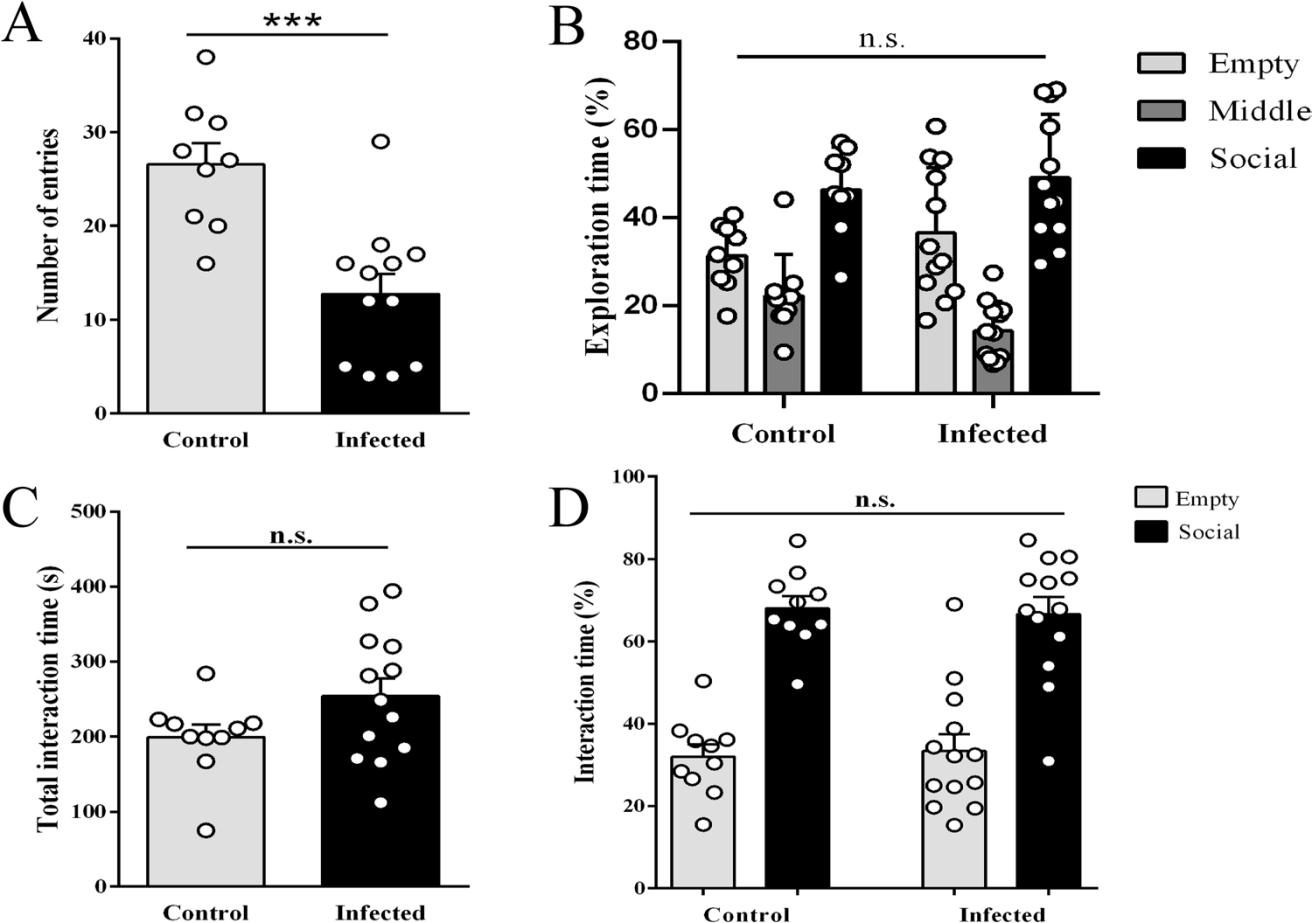
Chronic *T. gondii* infection did not alter social preference in mice. Infected mice had a reduced number of entries in chambers, indicating a decrease in locomotor activity during the test (**A**), but the percentual of exploration time in each chamber was similar between groups (**B**). Total interaction time with the cages was not significantly different between groups. All in-group differences were significant (*p* < 0.0001%). (**C**). Moreover, control and infected mice presented a significant preference to explore the social cage (**D**). Asterisks indicate statistically significant differences and N.S. not significance. ***p < 0.001. Symbols correspond to individual subjects. Control (n = 10), infected (n = 13).

The altered PPI response of infected animals prompted us to evaluate whether *T. gondii* affects other schizophrenia symptoms-related behavior in mice. In the three‐chamber social approach test, once more the motor dysfunction presented by infected mice was detected, expressed by a reduction in the total number of entries in the box chambers (Fig. 3A, 26 ± 2 control, 13 ± 2, *p* < 0.001). Nevertheless, we found no differences in percentual time exploration in each chamber between groups, which indicates that, despite severe motor deficits observed in the infected group, all subjects had the same pattern of exploration (Fig. 3B, percentage of total time spent in each chamber, control: 31 ± 7% empty chamber, 22 ± 9% middle chamber, 46 ± 9% social chamber; infected: 36 ± 14% empty chamber, 14 ± 6% middle chamber, 49 ± 14% social chamber, *p* = 0.144). Furthermore, control and infected animals did not show any difference in the total time of interaction (social + empty cages) (Fig. 3, 199 ± 17 s control, 253 ± 24 s infected, *p* = 0.095). These data clearly show that motor dysfunction did not impair mice's ability to explore the cages. Most importantly, both groups showed preference for interacting with the social cage rather than the empty cage (Fig. 3D, social cage exploration 68 ± 3% control, one-sample Student’s t-test against 50%, *p* < 0.001; 67 ± 4% infected one-sample Student’s t-test against 50%, *p* = 0.002). There was no statistically significant difference between control and infected mice regarding their social preference (*p* = 0.800).

### Chronic *T. gondii* infection decreased excitatory neurotransmitters levels in the prefrontal cortex and hippocampus

**Figure 4:**
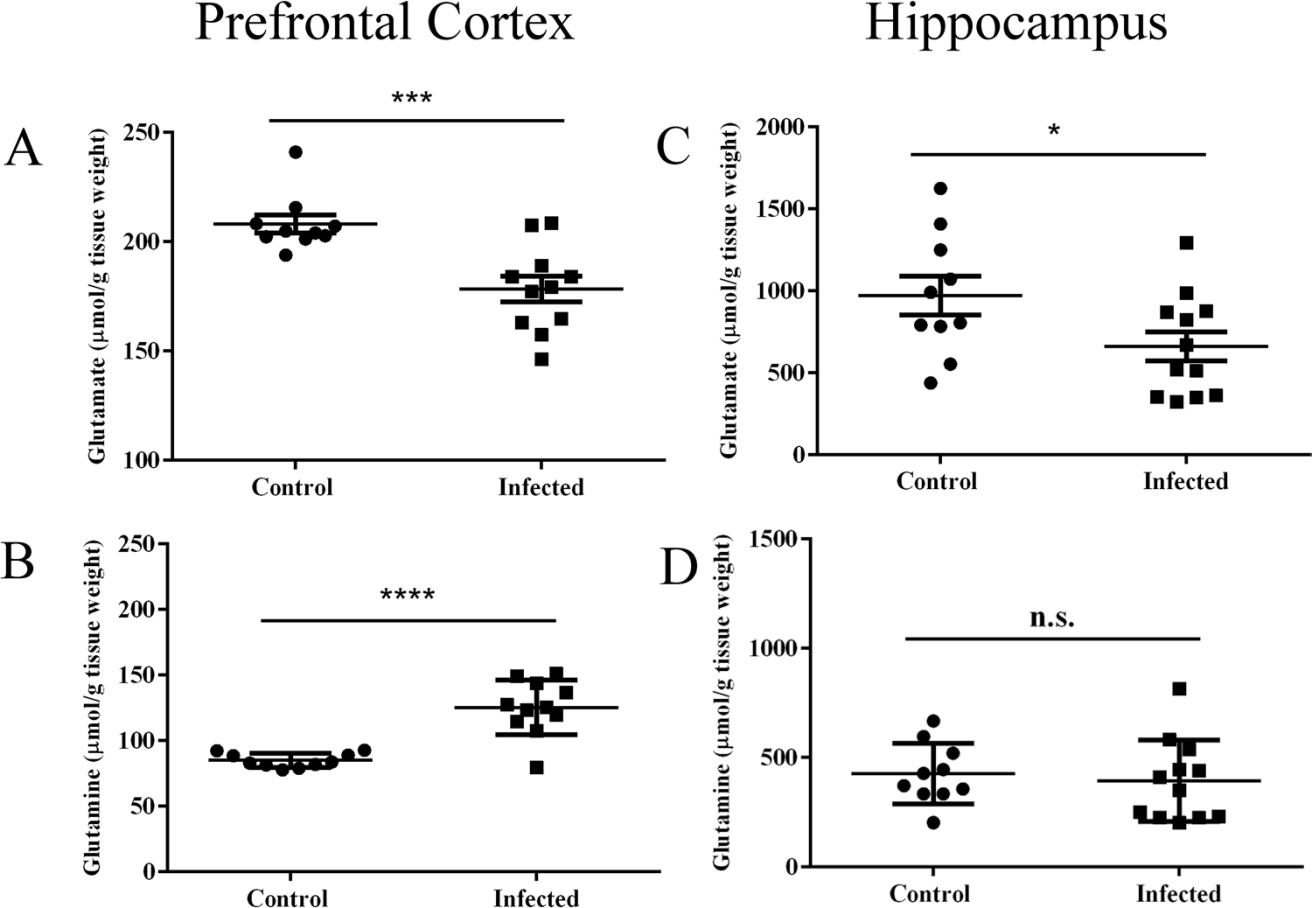
Reduction of glutamate in prefrontal cortex and hippocampus in *T. gondii* chronic infection. Glutamate was reduced in the prefrontal cortex (**A**) and hippocampus (**C**), while glutamine was increased in the prefrontal cortex (**B**) and unchanged in the hippocampus (**D**). Asterisks indicate statistically significant differences and N.S. not significance. *p < 0.05; ***p < 0.001 and ****p < 0.0001. Symbols correspond to individual subjects. Horizontal lines represent mean values for each group. Prefrontal cortex: control (n = 10), infected (n = 11). Hippocampus: control (n = 7), infected (n = 9).

We also evaluated whether *T. gondii* infection alters excitatory amino acid levels in the prefrontal cortex and hippocampus by using tissue homogenates. In the prefrontal cortex, we observed a significant reduction in glutamate levels (Fig. 4A, 208.2 ± 4.0 μM control, 178.3 ± 5.8 μM infected, *p* < 0.001), which was accompanied by an increase in glutamine levels (Fig. 4B, 85.0 ± 1.6 μM control, 125.3 ± 6.2 μM infected, *p* < 0.0001). In the hippocampus, glutamate was also reduced (Fig. 4C, 972.9 ± 118.3 μM control, 661.7 ± 89.6 μM infected, *p* < 0.05) but we did not detect any changes in glutamine levels (Fig. 4D, 425.9 ± 43.6 μM control, 393.0 ± 53.9 μM infected, *p* = 0.65).

**Figure 5:**
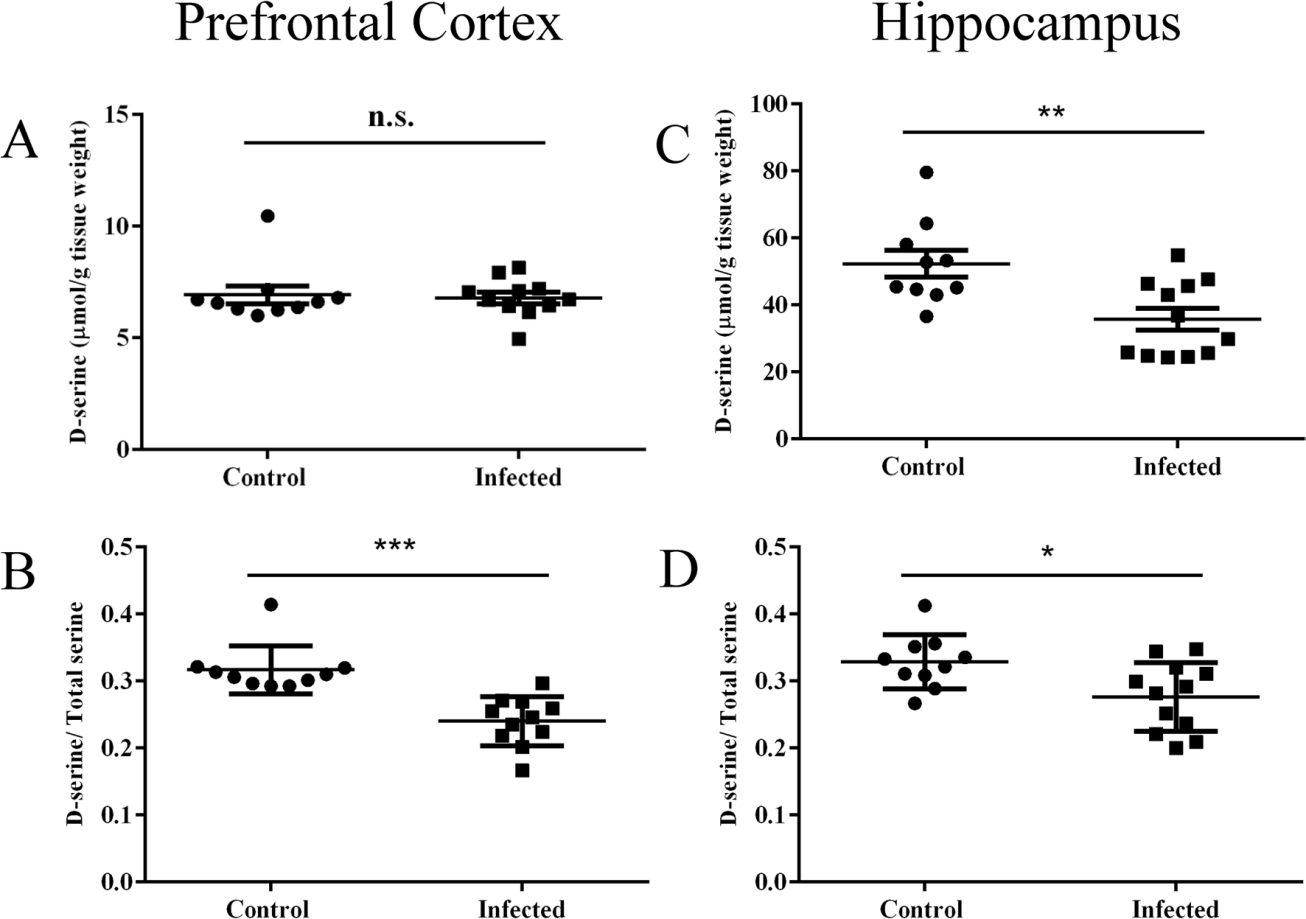
Reduction of D-serine in prefrontal cortex and hippocampus in *T. gondii* chronic infection. D-serine levels are reduced in the hippocampus (**C**) and unchanged in prefrontal cortex (**A**). D-serine/total serine ratio was reduced in both prefrontal cortex (**B**) and hippocampus (**D**). Asterisks indicate statistically significant differences and N.S. not significance. *p < 0.05, **p < 0.01; ***p < 0.001. Symbols correspond to individual subjects. Horizontal lines represent mean values for each group. Prefrontal cortex: control (n = 10), infected (n = 11). Hippocampus: control (n = 7), infected (n = 9).

Regarding D-serine levels, we did not observe any significant difference in total D-serine concentration between groups in the prefrontal cortex (Fig. 5A, 6.9 ± 0.4 μM control, 6.7 ± 0.2 μM infected, *p* = 0.78). However, we found a significant decrease in D-serine in the hippocampus (Fig. 5C, 52.3 ± 3.9 μM control, 35.7 ± 3.2 μM infected, *p* < 0.01). Beyond D-serine concentration, we also measured the D-serine / total serine ratio (D-serine / D + L-serine), which was significantly decreased in prefrontal cortex (Fig. 5B, 0.3 ± 0.01 μM control, 0.2 ± 0.01 μM infected, *p* < 0.05) and hippocampus (Fig. 5, 0.3 ± 0.01 μM control, 0.2 ± 0.01 μM infected, *p* < 0.001), indicating that there was a proportional reduction of D-serine in infected animals.

## Discussion

In this study, we aimed to investigate whether chronic *T. gondii* infection in C57Bl/6 mice with the cystogenic ME-49 strain induces behavioral and biochemical changes. As previously reported by us, mice presented a progressive weight loss and general locomotor activity deficits after infection^35–38^. Thus, this work described additional outcomes related to motor impairment in this *T. gondii* infection model. Furthermore, infected mice also showed a decrease in the startle response and neurotransmitter alterations in the prefrontal cortex and hippocampus compatible with neuropsychiatric disorders. However, we observed the preservation of social preference and working memory as shown in the social approach and the spontaneous alternation tasks, respectively. This selective disruption of behavioral and sensori-motor circuits suggests specific vulnerabilities to the chronic infection.

Infected mice did not lose the innate preference for social novelty, once exploration time of the social cage was superior to the time spent exploring the empty cage, in a similar way to control individuals. Although the locomotor deficits presented by the infected animals were detected in this task, reduced locomotion across the apparatus chambers was not translated in less interest to explore the cages since the total interaction time with both cages is comparable between groups. However, in a C57BL/6 mouse model of toxoplasmosis with cystogenic Prugniaud, female mice displayed impaired sociability and social memory only at the chronic phase of infection (8 weeks post-infection), with social preference preserved at the acute phase (3 weeks post-infection)^39^. In contrast, another study found that social interaction with the novel cage containing a strange mouse is higher in animals also infected with Prugniaud strain^40^. This longer duration of interaction with the social cage was also found in rats chronically infected with the highly virulent RH strain^41^. These apparent discrepancies between the studies might be attributed to the different chronification protocols and *T. gondii* strains used. Therefore, we propose that the chronic infection using type II ME-49 strain does not alter social preference, at least eight weeks post-infection.

In the tail suspension test, we observed that infected mice had a reduced tail suspension immobility time most likely associated with neurological damage^42^ that cannot be considered an attempt of escape. We also found that mice showed a strong tendency to turn to the same side in the spontaneous alternations task, as indicated by the increased position bias index. However, infected mice showed an increased percentage of spontaneous alternations compared to controls. This observation is in accordance with our group’s previous work, which showed that infected mice had standard spatial memory and novel object exploration time in the object placement test^37^. These findings indicate that, despite the motor deficits, their working memory processes are intact.

The acoustic startle response is a sensory-motor reflex that involves several brain and peripheral structures, being considered a good model for studying sensorimotor gating processes^43,44^. In line with our motor deficits data, *T. gondii* infected mice showed an important decrease in the startle amplitude, corroborating a possible neurological origin for the impairments described here. Moreover, differences in the basal startle reactivity between groups require extra caution when interpreting PPI data. Our data show that infected mice presented a blunted PPI response in comparison to control mice. Similar results were already reported using male Balb/C mice infected with the Prugniaud strain^45^. Despite that, others failed to identify any PPI change after infection^46,47^, and a PPI improvement was described for infected female Balb/C^48^. To the best of our knowledge, this is the first study to investigate the effects of chronic cerebral toxoplasmosis on sensorial reflex in the C57BL/6 strain, and the difference between our and previous data may be related to strain susceptibility to infection. However, our animals have a low basal startle amplitude, limiting the assay’s capacity to detect a further reduction in this response by prepulse presentation^49^. In fact, the relationship between startle amplitude and PPI is highly complex and often understated in animal studies. There is a clear correlation between PPI expression and startle reactivity that is stronger in subjects with low startle amplitude^50^, raising the assumption that PPI impairment showed by infected mice may be a consequence of their lower startle reactivity possibly caused by their motor deficits. The use of different pulse intensities to balance startle response between groups, or electrophysiological measures of the sensory gating, such as mismatch negativity or P20 potential, would clarify our findings.

Chronic *T. gondii* infection alters several neurotransmitter systems in the rodent brain, including glutamatergic synaptic homeostasis^12,29–32,51,52^. These alterations might be an outcome or even induce severe neuronal damage through programmed cell death and glutamate cytotoxicity^53^. Our results demonstrated that both glutamate and D-serine are reduced in the prefrontal cortex and hippocampus after chronic infection, which could be a sign of decreased glutamate turnover due to neurodegeneration, whereas neurotransmitter synthesis and release might be compromised. It is important to highlight that this reduction of glutamate and D-serine might be related to the startle impairment and possible PPI deficits observed in this study^54–56^.

All in all, we propose that chronic *T. gondii* ME-49 strain infection disrupts startle reflex and also glutamate and D-serine levels in C57Bl/6 mice, while social preference and working memory remain intact. In this context, it seems that some behavioral aspects are still preserved despite the severe brain damage and imbalanced neurotransmission in specific areas caused by chronic infection.

## Methods

### Animals

Female C57Bl/6 mice (postnatal day > 60) from Fundação Oswaldo Cruz (Fiocruz) breeding colony were used. Animals were group housed (maximum 5 per cage), in a 12/12 light–dark cycle room, with free access to standard food and tap water. All methods and procedures were carried out in compliance with the guidelines established by Colégio Brasileiro de Experimentação Animal (COBEA), the Guidelines on the Care and Use of Animals for Experimental Purposes and Infectious Agents (NACLAR) and the ARRIVE (Animal Research: Reporting of In Vivo Experiments) guidelines. All experimental protocols were approved by Fundação Oswaldo Cruz—Fiocruz Committee of Ethics for the Use of Animals (license L042/18 A1).

### Parasites and experimentally acquired toxoplasmosis

*T. gondii* of ME-49 strain (Type II) were used and maintained in C57BL/6 female mice, weighing about 12–18 g each. For infection, parasites were inoculated intraperitoneally with about 30 cysts/animals diluted in 200 μL of phosphate-buffered saline (PBS), while control animals were injected with PBS only. Animals were euthanized with ketamine the following day after behavioral tests. All the brains were partially collected for histopathology. Prefrontal cortices and hippocampi were dissected and homogenized to determine neurotransmitter levels as described below.

### Behavioral tests

Mice were behaviorally tested 8 weeks after infection using spontaneous alternations, tail suspension, prepulse inhibition of the startle reflex (PPI) and social approach tests. Behavioral tasks were performed during the light cycle, between 8:00 am and 5:00 pm in a dimly lit room with minimal background noise. All test apparatuses were cleaned with 10% ethanol between each session and with 70% ethanol at the end of the last session of experiments.

### Spontaneous alternations

Mice were allowed to freely explore a Y maze (40 × 8 × 20 cm each arm) for eight minutes. The total number and sequence of arms entrances were recorded. A spontaneous alternation is characterized when the animal enters three different arms in sequence. The percentage of spontaneous alternations was calculated as a measure of working memory integrity. The position bias index of each animal was calculated to investigate motor impairments' interference in the task^57^. This index indicates whether animals show a trend for always turning left or turning right in the maze, which might compromise results interpretation.

### Tail suspension test

The tail suspension test was conducted to investigate the development of depressive-like behavior. In this test, mice tend to develop an immobile posture when placed in an inescapably stressful situation after initial escape-oriented movements. Mice were individually suspended (using an adhesive tape placed 1 cm from the tip of the tail) about 65 cm above the floor for 6 min. Immobility was recorded only when mice hung passively and completely motionless. Depression-like behavior shown by the time spent immobile was measured in the last 4 min of the 6-min-long test as previously described^58^. Sessions were videotaped and the immobility time (s) was blindly scored.

### Prepulse inhibition of the startle reflex (PPI)

PPI test was performed to investigate the sensorimotor gating and was conducted as previously described^59^. Briefly, a startle box system (Panlab®) containing a sound generator and an accelerometer was used to record the amplitude of mice’s startle response (ASR). A white background noise (65 dB) was generated throughout the experiment. Mice were kept in the chamber for five minutes for habituation, and then five blocks containing a startle-inducing pulse (white noise, 120 dB, 50 ms) were presented. After that, mice were randomly exposed to 5 different blocks of stimuli (10 repetitions each): background noise, pulse (white noise, 120 dB, 50 ms), and pulse preceded by prepulse in three different intensities (72, 80 and 90 dB, white noise, 20 ms, 100 ms interstimulus interval). The interval between blocks was 20 ± 10 s. Mice ASR in each block was digitized and recorded (Startle v. 1.2.04, Panlab®). The percentage of startle reflex inhibition at different prepulse intensities was calculated using the following formula: %PPI = 100 − [100 × (ASR mean to prepulse + pulse trials/ASR mean to pulse alone trials)].

### Social approach task

Sociability assessment was conducted as previously described^60^, with minor modifications. A rectangular transparent plexiglass box (60 × 45 × 30 cm) divided into three equal compartments (20 × 45 × 30 cm) was used. Openings between compartments allow the animal to move from one compartment to another freely. Cylindrical aluminum cages (9.5 cm high × 8 cm diameter) were used to contain stimuli mice. Test mice were habituated in the three-chamber apparatus in the presence of empty cages, one in each lateral chamber, for 15 min one day before the test. On the same day, social stimuli (conspecific adult female mice) were habituated inside cylindrical cages for two sessions of 30 min. In the social approach test, a social stimulus was placed inside a cage in one of the lateral compartments while an identical empty cage was placed in the opposite compartment. Test mice were initially placed in the central compartment without access to the lateral compartments for 5 min. After that, blocking walls were removed and test mice could move between compartments for 10 min. Sessions were videotaped and interaction time with the both empty and social cages (s), permanence time and the number of entries in each compartment were analyzed.

### Measurement of aminoacids levels

After the infection period, animals were euthanized, brains were removed and the prefrontal cortex and hippocampus of each animal were rapidly dissected. Tissue fragments were homogenized at 4 °C in RIPA buffer (Sigma, St. Louis, USA). We analyzed glutamate, l-serine, glutamine, and D-serine levels by high-performance liquid chromatography (HPLC) as previously described^61,62^.

### Histopathology

The brains were fixed in 5% formalin and embedded in paraffin. Serial coronal 20 µm thick sections were cut and slides were routinely stained by hematoxylin and eosin (HE) for histopathological evaluation. Brain samples were evaluated for the severity and distribution of inflammation and the presence of *T. gondii* tissue cysts.

### Statistical analysis

SigmaStat version 3.01 (Jandel Scientific Corporation®) or GraphPad Prism® version 6.01 softwares were used. Unpaired Student’s t-test was performed to analyze data between experimental groups (control and infected). In the social approach test, the percentage of interaction time with the social cages was analyzed using a one-sample Student's t-test using a 50% value as standard, while the data that contains the percentage of time spent in each chamber of the apparatus was analyzed by two-way ANOVA followed by Bonferroni-Sídák test for multiple comparisons. % PPI data were analyzed by two-way repeated-measures ANOVA with experimental groups as the first factor and prepulse intensity as the second one. This analysis was followed by Tukey’s test for multiple comparisons. Significance level *p* < 0.05 was considered significant. All experimenters were blind to control and experimental groups.

## Data Availability

The datasets generated during and/or analysed during the current study are available from the corresponding author on reasonable request.
